# Effectiveness of a pre-adolescent inter-generational intervention to address HIV and obesity in South Africa, using a pretest-posttest design

**DOI:** 10.1186/s12889-021-12228-z

**Published:** 2021-12-11

**Authors:** Keshni Arthur, Nicola Christofides, Gill Nelson

**Affiliations:** School of Public Health, University of the Witwatersrand, 27 St Andrews Road, Parktown, Johannesburg, 2193 South Africa

**Keywords:** Pre-adolescent, Parent, Nutrition, Physical activity, Communication

## Abstract

**Background:**

Strengthening pre-adolescents knowledge and skills through an age- and culturally-appropriate intervention could prevent health issues later in life. Early interventions could influence the trajectory of future risky behaviour, and may influence health behaviour amongst their parents. *The CIrCLE of Life Initiative* was developed to address HIV and obesity. We evaluated whether the combined intervention increased knowledge, enhanced skills, and/or promoted healthy behaviour among students (9–12 years old) and their parents.

**Methods:**

The study was conducted from May to December 2018. Trained educators delivered 30-min lessons over ten consecutive weeks with 537 Grade 6 students at five government-run schools, in a district, in South Africa. Schools were purposively selected based on socioeconomic status and urban-rural classification. Students communicated with parents through shared homework activities. A pretest-posttest study design was used, with a 3-month follow up. Both groups completed self-administered paper-based questionnaires. A score of subscales was used in analysis. The pretest and posttest scores were compared for students and parents using a dependent t-test. Differences in outcomes by school quintile were compared using one-way ANOVA.

**Results:**

Response rates were high for both students (80.6%) and their parents (83.4%). Statistically significant differences were observed in HIV knowledge in students pretest (mean 8.04, SD 3.10) and posttest scores (mean 10.1, SD 2.70; *p* < 0.01), and their parents (mean 10.32, SD 2.80 vs 11.0, SD 2.50; *p* < 0.01). For both students and parents, pre- and post-test obesity awareness mean scores were similar, 1.93, SD 0.92 and 2.78, SD 0.57; *p* < 0.01, for students; and 2.47, SD 0.82 and 2.81, SD 0.54; p < 0.01, for parents. In the posttest, statistically significant changes were also observed in both groups, enhancing skills in measuring body mass index and pulse rate, and interpreting food labels. Students had a high intention to share gained knowledge with parents who had a high intention to receive it (89.4 and 89.5%, respectively).

**Conclusion:**

The intervention increased knowledge about HIV and obesity-related awareness, and it enhanced skills in selected outcomes among pre-adolescents and parents. Accurate messages and enhanced communication skills could support inter-generational knowledge transfer.

**Trial registration:**

ClinicalTrials.gov Identifier: NCT04307966 retrospectively registered on 12 March 2020.

**Supplementary Information:**

The online version contains supplementary material available at 10.1186/s12889-021-12228-z.

## Background

South Africa, like other developing countries, is in the midst of a health transition that is characterised by the double burden of communicable disease, such as human immunodeficiency virus (HIV) [1], and non-communicable disease (NCD) such as cardiovascular disease [2]. Obesity is a key contributor to the rise in the prevalence of NCDs [3, 4] and, together with HIV/AIDS, is a cause of early mortality [1]. The burden of obesity and HIV affects both adults and children. Based on the body mass index (BMI) score, 68% of women and 31% of men (15 years and older) are overweight (BMI ≥ 25 kg/m2) or obese (BMI ≥ 30 kg/m2) [5]. Based on weight-for-height measures, 13% of children under the age of 5 years are overweight; a prevalence of more than twice the global average of 6.1% [5]. Approximately 7.5 million South Africans of all ages are living with HIV [6]. HIV prevalence among adults (15 to 49 years) is 19.0%; 25.0% among females and 12.9% among males. It is estimated that 340,000 children (0 to 14 years) are HIV infected [6].

Increasing global obesity rates have resulted in the development of interventions, aimed at children and adolescents, to stem the epidemic [7–12]. Many of these evidence-based programmes aim to reduce BMI through decreasing sedentary behaviour, influencing dietary behaviour, or combinations of the two, but have yielded mixed results [7–10]. Some have shown improvement in health behaviour (increase in physical activity, healthier diets) and in nutrition-related knowledge [9, 10]. Other interventions noted minimal changes in anthropometry [7, 8]. A South African nutrition and physical activity intervention, ‘Healthkick’, did not significantly improve the quality of diet in children because the intervention model was not a suitable fit for the low-income setting [13].

In the past decade, HIV programmes have focused on improving access to antiretroviral treatment, adherence and prevention efforts to reduce risk factors. Risky sexual behaviours associated with HIV infection among young people include early sexual debut, multiple partners, gender and social norms, forced sex and lack of condom use [14]. A review of the effectiveness of HIV/AIDS prevention interventions in sub-Saharan Africa found that school-based, adult-led, curriculum-based interventions were effective in reducing reported risky sexual behaviour [15]. There are limited interventions targeting adolescents, who are at highest risk and highest prevalence settings [16]. South African HIV prevention initiatives have shown positive outcomes through various media platforms and programmes designed for youth [17] and a multi-media vehicle that demonstrated a positive impact on certain risky sexual behaviours (condom use knowledge, attitudes towards people with HIV/AIDS and delaying sex) [18, 19].

HIV and obesity, are often stand-alone initiatives and infrequently combined, but are often directed at the same populations and comprise related activities. For example, HIV interventions often address nutrition related to living positively, while interventions directed at reducing over-nutrition to prevent obesity may have similar messages [7, 10, 16, 20]. Managing potential health risks at an early age [21], and improving the quality and quantity of communication between parent and child [22, 23], are ways of approaching a combined intervention to address the overlap between HIV and obesity.

Pre-adolescence (9 and 12 years) [24] could serve as an important developmental stage to address knowledge and skills. Early interventions can successfully lead to longer-term behaviour change [18, 25, 26]. For example, a childhood HIV intervention, implemented between 2004 and 2008, was associated with a decrease in young women’s HIV risk and suggested an impact on key risky sexual behaviours, approximately 10 years later [18]. Young people receive the most protection against risky behaviour with early and repeated exposure to interventions [26].

Interventions targeting pre-adolescents could also influence health behaviours amongst their parents/caregivers upon whom they are most dependent for their health needs and well-being [21]. In this paper, the term ‘parent’ also denotes primary caregiver, unless otherwise stated. The conventional view is that parents teach their children, inculcating their knowledge, values and beliefs [21]. Parents are uniquely positioned to fulfil the critical role of shaping adolescent behaviour through their parenting practices and parent-child communication [27]. Several evidence-based practices aimed to increase the quality and quantity of parent-adolescent communication, to prevent HIV in adolescents [22, 23], and have included strategies such as, communication about sexual risk and HIV prevention and comfort with communication. The bi-directional influence between parents and children is also possible [28]; however, studies on the impact of a child’s influence on parent’s knowledge, attitudes and behaviours remain limited with inconclusive findings.

*The CIrCLE of Life Initiative* was developed to increase awareness about HIV and obesity risks and prevention [29]. The programme supports knowledge transfer directly to pre-adolescent students, and indirectly to their parents, for immediate- and long-term benefits. The purpose of this study was to evaluate whether the intervention increased knowledge, enhanced skills and/or promoted healthy behaviour among pre-adolescent students and their parents.

## Methods

### Study design

This was a quasi-experimental pretest-posttest outcome evaluation of *The CIrCLE of Life Initiative*. The study was registered with ClinicalTrials.gov (NCT04307966) on March 12, 2020. The delay in registration was due to stakeholder engagement in the intervention design process. The authors confirm that all ongoing and related trials for this intervention are registered. A Transparent Reporting of Evaluations with Nonrandomized Designs (TREND) Statement Checklist [30] is presented in Additional file 1.

### Participants

The intervention was rolled out to 537 Grade 6 students (and one of their parents) at five government-run primary schools in a district located in Gauteng province, South Africa. The schools were purposively selected based on quintile and urban-rural classification. Urban and rural schools in South Africa are categorised into five quintiles [31], based on the relative wealth of their surrounding communities. Those in the most impoverished communities are classified as Quintile 1 schools and those serving the wealthiest communities, as Quintile 5 schools. Quintile 1, 2 and 3 schools do not charge fees. The government subsidises schools financially, with Quintile 1 schools receiving the highest allocation per student [31]. Two schools were classified as Quintile 1, and one each as Quintile 2, 3 and 4. There were no Quintile 5 schools in the district. The Quintile 4 school was the only fee-paying school and the only school that did not provide a nutrition programme (subsidised meals provided by the school). Three of the schools were in rural areas and two were in urban areas. The student complement per school ranged from 650 to 1100. There were approximately 100 Grade 6 students in each school. None of the schools had computer technology and internet access for students.

Seven educators were recruited to implement the intervention to all 12 Grade 6 classes. The number of educators per school ranged from 25 to 30. The educator-student ratio per grade was highest in a Quintile 1 rural school with 54 students to one educator (54:1); the lowest ratio was in the other Quintile 1 rural school (39:1).

### Overview of the intervention

**Fig. 1:**
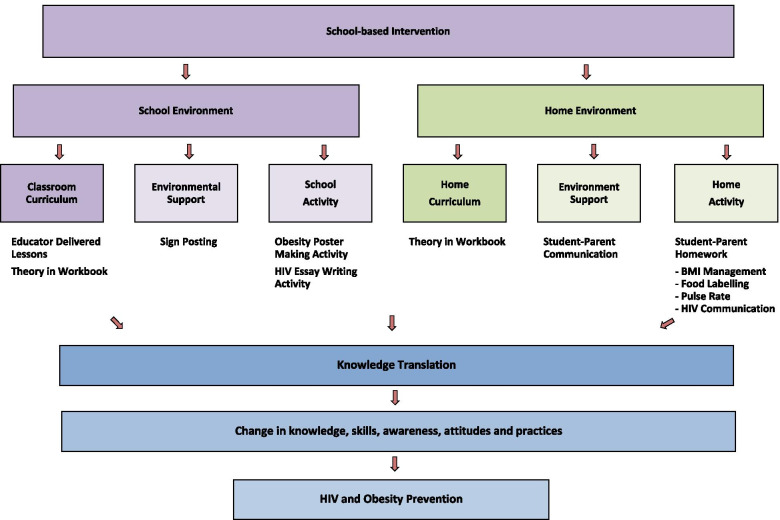
The CIrCLE of Life Initiative. A detailed overview of the intervention, *The CIrCLE* (Child Influencing paRent Communication for Life Education) *of Life Initiative*. Reprinted from Arthur et al. (2020) [29], under a CC by licence, with permission from Health Education Journal, original copyright 2020 [29]

*The CIrCLE* (Child Influencing paRent Communication for Life Education) *of Life Initiative* (Fig. 1) had both school and home components, each comprising a learning curriculum, environmental support, and activities that aimed to increase student knowledge and skills, and to engage parents [29]. The intervention (lesson content and material) was age- and culturally-appropriate and was acceptable to both pre-adolescents and adults [29]. Examples included eating food from different cultures, and talking to children about sex-related matters. Trained educators delivered lessons about HIV and obesity to all Grade 6 students at the schools. The classroom curriculum for students required delivery of a five-hour face-to-face intervention delivered weekly through ten 30-min lessons. Students were asked to communicate their learnings to their parents at home. Parents were requested to read through the lesson in a workbook and to sign acknowledgement that they had read it. The workbook contained shared student-parent homework activities that took approximately 30 min per week to complete.

The intervention targeted three key behaviours: (1) modifying diet, (2) increasing physical activity and (3) increasing student–parent communication to decrease HIV-related risk behaviours through: (a) information dissemination, (b) translation of knowledge into skills through practice and (c) strengthening autonomous support and self-efficacy in communication. Specific behaviour change theory, based on the taxonomy of behaviour change [33], included advance organisers (theories of information processing) to increase HIV- and obesity-related knowledge, guided practice (social cognitive theory and theories of self-regulation) to enhance change skills, and self-efficacy in communication.

Educator were familiar with the content as these were included in the national school curriculum (Table 1). *The CIrCLE of Life* curriculum was designed to fit in with the national school curriculum. The communication aspects and the shared homework activities were new approaches.

**Table.1:** Overview of lesson content

Lesson	Content	Skills	Reinforcing Activity	Parent-Student Homework
**One:** Obesity	Healthy body image^a^ Underweight, overweight and obesity Body Mass Index (BMI)	How to measure weight How to measure height Interpreting BMI	Diary of a healthy kid Crossword puzzle	Measure and interpret student’s BMI
**Two:** Nutrition	Healthy eating^a^ Food-based dietary guidelines^a^	How to interpret a food label^a^	Word search puzzle Colouring-in picture	Interpret a food label at home
**Three:** Physical Activity	Benefits of physical activity Heart rate (pulse)	Measuring and interpreting pulse	Colouring-in picture	Measure and interpret family member’s pulse Obesity poster activity
**Four:** HIV/AIDS	HIV/AIDS^a^ Understanding the risks of HIV/AIDS	Reducing the risk of HIV/AIDS	Crossword puzzle	HIV/AIDS story-writing activity
**Five:** Communicating about HIV/AIDS	Communication Importance of communication Healthy ways of communicating	Dealing with discomfort and support for sexual-specific communication	Communication game	Family communication exercise

Reprinted from Arthur et al. (2020) [29], under a CC licence, with permission from Health Education Journal, original copyright 2020 [29]

^a^Already a part of the existing Grade 6 national school curriculum

### Procedures

Prior to intervention delivery, a needs assessment [29] and an organisational readiness evaluation [32] were conducted to ensure that the intervention would be well received by the target population and suitably implemented. The needs assessment confirmed that HIV was a concern in the communities and that some students experienced malnutrition. It led to adaptations to suit the context of implementation. Organisational readiness assessments identified potential barriers and contextual factors that needed to be addressed during implementation [32]. It was also used to gain educators’ support for the intervention. All educators indicated that the intervention was a fit for their schools, regardless of location or socio-economic status.

A process evaluation was conducted alongside the pretest-posttest outcome evaluation. Although both evaluations ran concurrently, the results were analysed and reported separately. All process evaluation data were analysed before the outcome evaluation results were available, to minimise the risk of bias in interpretation [34]. The process evaluation showed that the contexts of the different schools varied.

The intervention was implemented during the 2018 school academic year to all Grade 6 students. Data were collected using self-administered questionnaires (pretest and posttest for students and parents). The pretest data were collected in May 2018. The intervention was implemented from June to August 2018. The posttest data collection was conducted 3 months later.

Ethical approval was obtained from the Human Research Ethics Committee of the University of the Witwatersrand (clearance certificate no. M180220). The Gauteng Department of Education provided permission to conduct the study in the selected schools. School principals provided permission to conduct the study. Written informed consent and assent to participate in the study were obtained from the participating parents and students, respectively. A distress protocol was put into place to address any issues that arose among students related to the topics.

### Measures

The primary outcomes were students’ and parents’ knowledge, attitude and skills, comparing pretest and posttest scores. The 69-item student pretest questionnaire and 67-item student posttest questionnaire measured: socio-demographic characteristics (6 items); HIV/AIDS-related knowledge, attitude, behaviour, practices and skills (21 items); obesity-related awareness, attitudes and skills (6 items); nutrition-related awareness, attitudes, behaviour and skills (14 items); physical activity-related awareness, attitudes, behaviour and skills (7 items); cigarette and alcohol behaviour (4 items); child-parent communication (8 items); and general comments (3 items). Socio-demographic items were excluded from the posttest. Additional questions such as “What did you like/dislike about the programme?”, were added. All other questions were the same as the pretest.

Questionnaire design was based on various publicly accessible survey tools [35, 36]. All HIV/AIDS knowledge items were summed to create a score that measured knowledge about transmission and associated risk factors. A Cronbach’s alpha was calculated to assess the internal consistency of the overall HIV knowledge score and, at 0.73, indicated acceptable internal consistency. Attitude items measured openness about status, for example, “If a family member has HIV, would you want to keep it a secret?”. An example of a question that assessed perception of stigma in the community was, “Do people in your community stay away from people who have HIV?”. An example of a question that assessed the exposure to HIV/AIDS education was, “Have you ever been taught about AIDS or HIV infection at school?”. Responses were measured using a Likert scale: (1 = no, 2 = unsure, 3 = yes).

Obesity-related awareness, attitude and skills (BMI calculation and interpretation) were also explored. An example of a question that assessed BMI awareness was, “Have you heard of body mass index?”. Responses were measured on the same Likert.

The nutrition questions were based on survey tools such as the South African National Health, Demographic and Behaviour Survey, 2011 [37] and the South African National Health and Nutrition Examination Survey, 2011/12 [38]. The nutrition section investigated awareness, attitudes, behaviours and skills (interpreting a food label). An example of a question that assessed importance of nutrition to health was, “How important is what you eat to your health?”. Responses were based on a 5-point Likert scale (1 = not important, to 5 = very important).

The physical activity section explored awareness, attitudes, behaviours and skills. An example of a question that assessed attitude to the importance of physical activity for health was, “How important is physical activity to your health?”. Responses were based on a 5-point Likert scale (1 = not important, to 5 = very important). The physical activity measures were based on the intervention content and were not validated.

The child-parent communication section evaluated behaviour and self-efficacy in communication. The tool was adapted from an existing self-efficacy in communication tool [39]. An example of a question that assessed communication was, “How easy is it to talk to your parents/guardian about HIV and AIDS?”. Responses were based on the same 5-point Likert scale already described. The responses to behaviour in communicating about adolescent issues section were combined into one score. A Cronbach’s alpha was calculated to assess the internal consistency of the overall score and, at 0.73, indicated acceptable internal consistency.

A final, general section enquired if the students intended to transfer knowledge to their parents or change their behaviours.

The 76-item parent pretest and 66-item posttest questionnaires elicited parents’ feedback. Questions were similar to those in the student questionnaire, with eight additional questions on socio-demographics. The Cronbach’s alpha was 0.78 for the HIV knowledge score and 0.77 for the communication score in the parent pretest, indicating acceptable internal consistencies.

The questionnaires were piloted in a group of 40 participants (20 students and 20 parents) from other schools in the district, and led to some amendments.

### Data analysis

Data from the questionnaires were captured using REDCap 9.3.1 [40]. Data were imported into Stata 14 for statistical analysis. All data were recoded to categorical variables based on the distribution of the data. A targeted aim of the intervention was to shift from a negative to a more positive behaviour. Demographic characteristics were described using frequencies and proportions. The analysis adjusted for the cluster sampling (schools), using the svy Stata command. A score for domains (subscales) was created for analysis. The pretest scores (SD) were compared to the posttest scores (SD) for students and parents. A dependent t-test was used to assess differences in mean scores from pretest to posttest. A one-way ANOVA was conducted to compare the student outcomes of the intervention between the school quintiles. Parents were excluded from this analysis because we hypothesised that school quintiles could directly affect students. In all analyses, differences between quintiles were considered to be statistically significant at the 95% level. The Bonferroni ad hoc test assessed which pairings of quintiles were driving the statistically significant differences.

## Results

### Baseline characteristics of participants

**Fig. 2:**
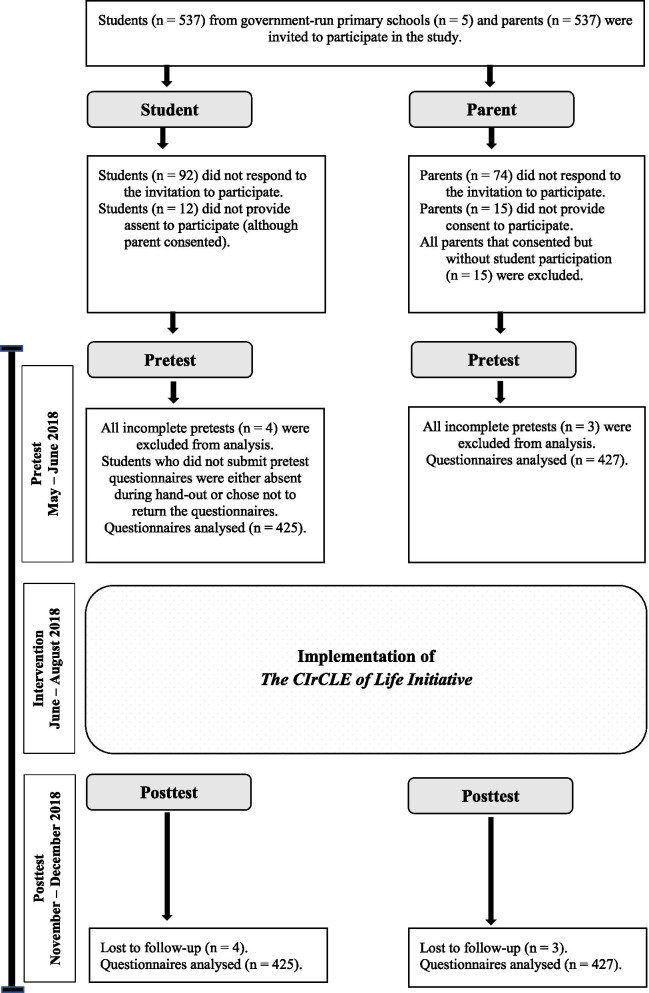
Flow diagram illustrating student and parent participation, with timelines. A detailed overview of student and parent participation, and timelines of the intervention

The response rates were high: 433 of the 537 students (80.6%) and 448 of the 537 parents (83.4%) participated. For the analysis, however, only data from students and parents who completed both pre- and post-test questionnaires were included, i.e. 425 students and 427 parents (Fig. 2).

The baseline characteristics and risk behaviours of the study participants (students and parents) who completed both the pre- and post-tests are shown in Table 2. The mean age of students was 11.9 years (SD 0.8); 230 (54.1%) were female. The mean age of the parents who responded was 40.7 years (SD 10.3); 85.5% were female. A similar percentage of students reported living with both parents and a single parent (44.2 and 42.1%). Although 13.7% of students reported that they lived with caregivers other than biological parents, e.g. in an orphanage or with a grandparent/extended family member, the parents’ response, however, showed this figure to be higher, at 19.9%.

**Table.2:** The baseline characteristics and risk behaviours of the students (*n* = 425) and parents (*n* = 427)

Variable	n	%
**Student**		
Sex		
Male	195	45.9
Female	230	54.1
School		
A	94	22.1
B	73	17.2
C	69	16.2
D	115	27.1
E	74	17.4
School quintile		
1	167	39.3
2	70	16.5
3	114	26.8
4	74	17.4
School location		
Rural	236	55.5
Urban	189	44.5
Lives with		
Both parents	188	44.2
Mother only	157	36.9
Father only	22	5.2
Caregiver	58	13.7
Transportation mode to school		
Walk	192	45.2
Bus	116	27.3
Taxi	77	18.1
Car	38	8.9
Other	2	0.5
Travel time to school (minutes)		
5–20	188	44.2
21–40	80	18.8
41–60	17	4.0
> 60	18	4.2
Uncertain	122	28.7
Cigarette smoking behaviour		
Everyday	23	5.4
A few times every week	5	1.2
A few times during the entire month	15	3.5
Only once in the past month	12	2.8
Never	370	87.1
Alcohol consumption behaviour		
Everyday	15	3.5
A few times every week	16	3.8
A few times during the entire month	22	5.2
Only once in the past month	44	10.4
Never	328	77.2
HIV testing behaviour		
Not tested/uncertain	294	69.2
Attitude about weight		
Unhappy with current weight	67	15.8
**Parent**		
Parent	342	80.1
Caregiver	85	19.9
Sex		
Male	62	14.5
Female	365	85.5
Level of education		
Did not complete Grade 12	225	53.2
Grade 12	126	29.8
Post-Grade 12 qualification	72	17.0
Employment status		
Currently employed	137	32.9
Spouse currently employed	106	24.8
Government Social Grant dependent		
Child Grant	280	65.6
Old Age Pension Grant	22	5.2
Foster Child Grant	20	4.7
Disability Grant	6	1.4
Unemployment Income Fund	1	0.2
Cigarette smoking behaviour		
Everyday	32	7.5
A few times every week	7	1.6
A few times during the entire month	1	0.2
Only once in the past month	9	2.1
Never	378	88.5
Alcohol consumption behaviour		
Everyday	14	3.3
A few times every week	31	7.3
A few times during the entire month	50	11.7
Only once in the past month	64	15.0
Never	268	62.8
HIV testing behaviour		
Not tested	129	30.2
Attitude about weight		
Unhappy with current weight	107	25.1

A little over half (57.7%) of the parents stated that they were formally employed, while others, including some in formal employment, relied on government grants to support their families. Overall, 65.6% of parents received government child support grants. While 53.2% of parents had not completed high school, 17.0% of parents had a post-school qualification.

Nearly half of the 192 students walked to school (45.2%), indicating some physical activity. In the pretest survey, most participants (84.2% of students and 74.9% of parents) indicated that they were happy with their weight.

Fifty-five (12.9%) students and 49 (11.5%) parents reported smoking cigarettes in the past month, while 97 (22.9%) students and 159 (37.2%) parents reported consuming alcohol in the past month. More parents than students had reported being tested for HIV, with 298 (69.8%) parents and 131 (30.8%) students. Differences in pretest and posttest scores for students and parents on HIV/AIDS, obesity, nutrition, physical activity and communication subscales are presented in Table 3.

**Table 3:** Differences in students (*N* = 425) and their parents (*N* = 427) pretest and posttest scores on HIV/AIDS, obesity, nutrition, physical activity and communication subscales

Subscale	Student			Parent		
	Pretest (***N*** = 425) Mean (SD)	Posttest (***N*** = 425) Mean (SD)	***P***-value	Pretest (***N*** = 427) Mean (SD)	Posttest (***N*** = 427) Mean (SD)	***P***-value
**HIV/AIDS**						
Knowledge about HIV/AIDS	8.04 (3.10)	10.1 (2.70)	< 0.01^*^	10.32 (2.80)	11.0 (2.50)	< 0.01^*^
Attitude to openness about status	1.85 (0.95)	1.92 (0.95)	0.21	1.94 (0.93)	2.03 (0.94)	0.09
Perception of stigma in community	2.19 (0.83)	2.25 (0.79)	< 0.01^*^	2.44 (0.78)	2.43 (0.79)	0.69
Perceived risk about contracting HIV/AIDS	1.98 (0.86)	1.93 (0.86)	0.31	1.73 (0.86)	1.74 (0.86)	0.96
Exposure to HIV/AIDS education	2.36 (0.87)	2.84 (0.50)	< 0.01^*^			
Behavioural intention to test for HIV				2.64 (0.71)	2.70 (0.64)	0.11
**Obesity**						
Awareness about obesity	1.93 (0.92)	2.78 (0.57)	< 0.01^*^	2.47 (0.82)	2.81 (0.54)	< 0.01^*^
Awareness about BMI	1.40 (0.68)	2.56 (0.75)	< 0.01^*^	1.87 (0.91)	2.61 (0.72)	< 0.01^*^
Skill (Measuring and interpreting BMI)	1.38 (0.66)	2.32 (0.86)	< 0.01^*^	1.69 (0.88)	2.40 (0.83)	< 0.01^*^
**Nutrition**						
Perceived importance of nutrition to health	2.65 (0.65)	2.62 (0.69)	0.41	2.67 (0.62)	2.67 (0.65)	0.41
Awareness about food labels	2.15 (0.88)	2.83 (0.50)	< 0.01^*^	2.25 (0.88)	2.81 (0.52)	< 0.01^*^
Skill (Interpreting a food label)	2.02 (0.90)	2.78 (0.56)	< 0.01^*^	2.21 (0.88)	2.76 (0.58)	< 0.01^*^
**Physical Activity**						
Attitude to importance of physical activity for health	4.44 (1.03)	4.45 (1.04)	0.94	4.34 (1.02)	4.47 (0.98)	0.03
Awareness about pulse rate	1.60 (0.82)	2.59 (0.76)	< 0.01^*^	2.17 (0.91)	2.74 (0.60)	< 0.01^*^
Skill (Measuring pulse rate)	1.42 (0.70)	2.43 (0.81)	< 0.01^*^	1.82 (0.89)	2.44 (0.80)	< 0.01^*^
**Communication**						
Behaviour in communicating about adolescent issues	16.5 (4.9)	16.7 (4.8)	0.42	16.5 (5.0)	17.1 (5.0)	0.02^*^

* Statistically significant

### Pretest-posttest HIV results

In the HIV/AIDS knowledge subscale, statistically significant differences were observed for students, with an increase from pretest (mean 8.04, SD 3.10) to posttest scores (mean 10.1, SD 2.70; *p* < 0.01). Similar results were observed in parents, with an increase from the pretest (mean 10.32, SD 2.80) to the posttest scores (mean 11.0, SD 2.50; *p* < 0.01).

Other subscales that reached statistical significance were observed only in students, in perception of stigma in the community (*p* < 0.01) and exposure to HIV/AIDS education (*p* < 0.01).

### Pretest-posttest obesity results

Statistically significant differences were observed in all obesity subscales for both students and parents. In the awareness about obesity subscale, there was an increase between the pretest mean scores (mean 1.93, SD 0.92) and posttest scores (mean 2.78, SD 0.57; *p* < 0.01). Similar results were observed in parents, between the pretest (mean 2.47, SD 0.82) and posttest scores (mean 2.81, SD 0.54; *p* < 0.01).

### Pretest-posttest nutrition results

Statistically significant differences in subscales awareness about food labels and skill in interpreting a food label, for both students and parents (Table 3). There were no differences in the subscale measuring the importance of nutrition to health.

### Pretest-posttest physical activity results

With the exception of attitude towards importance of physical activity for health, all other physical activity subscales showed statistically significant differences for both students and parents.

### Pretest-posttest communication results

No statistically significant differences were observed for students regarding communicating about adolescent issues with their parents (Table 3) (16.5, SD 4.9 vs 16.7, SD 4.8). However, statistically significant differences were observed in parents, between the pretest (mean 16.5, SD 5.0) and the posttest communication scores (mean 17.1, SD 5.0; *p* = 0.02).

In the posttest survey, 89.4% of students indicated that, if the programme continued, they would have a high intention to share the knowledge gained with their parents. In the posttest, 89.5% of parents indicated that, if the programme continued, that they would have a high intention to receive the knowledge gained from students.

Differences in students pretest and posttest scores were observed for HIV/AIDS, obesity, nutrition, physical activity and communication subscales by school quintile (Table 4).

**Table 4:** Differences in students (*N* = 425) pretest and posttest scores on HIV/AIDS, obesity, nutrition, physical activity and communication subscales by school quintile

Subscale	Quintile	Pretest			Posttest		
		Mean (SD)	F Statistic	***P***-value	Mean (SD)	F Statistic	***P***-value
**HIV/AIDS**							
Knowledge about HIV/AIDS	1	7.95 (3.07)	10.31	< 0.01^*^	9.68 (2.76)	13.15	< 0.01^*^
	2	7.46 (3.25)			10.21 (2.58)		
	3	7.43 (3.08)			9.70 (2.98)		
	4	9.76 (2.68)			11.82 (1.32)		
Attitude to openness about status	1	1.92 (0.95)	2.41	0.07	1.96 (0.96)	1.31	0.27
	2	1.77 (0.94)			1.89 (0.94)		
	3	1.96 (0.97)			2.01 (0.96)		
	4	1.62 (0.89)			1.74 (0.91)		
Perception of stigma in community	1	2.25 (0.81)	4.23	< 0.01^*^	2.31 (0.79)	0.22	0.89
	2	1.96 (0.79)			2.30 (0.79)		
	3	2.03 (0.90)			2.32 (0.80)		
	4	2.34 (0.73)			2.23 (0.80)		
Perceived risk about contracting HIV/AIDS	1	1.98 (0.85)	0.46	0.71	1.88 (0.87)	1.96	0.12
	2	2.06 (0.87)			1.89 (0.83)		
	3	2.00 (0.86)			2.10 (0.85)		
	4	1.89 (0.88)			1.84 (0.88)		
Exposure to HIV/AIDS education	1	2.36 (0.89)	6.28	< 0.01^*^	2.77 (0.57)	3.13	0.03
	2	2.24 (0.88)			2.94 (0.29)		
	3	2.24 (0.91)			2.80 (0.56)		
	4	2.74 (0.64)			2.95 (0.33)		
**Obesity**							
Awareness of Obesity	1	1.89 (0.93)	2.98	0.03	2.65 (0.70)	6.68	< 0.01^*^
	2	1.80 (0.92)			2.94 (0.29)		
	3	1.90 (0.90)			2.75 (0.60)		
	4	2.22 (0.91)			2.93 (0.30)		
Awareness about BMI	1	1.38 (0.69)	0.09	0.97	2.52 (0.77)	6.60	< 0.01^*^
	2	1.41 (0.63)			2.74 (0.58)		
	3	1.41 (0.68)			2.36 (0.85)		
	4	1.41 (0.70)			2.78 (0.58)		
Skill (Measuring and interpreting BMI)	1	1.36 (0.67)	0.60	0.62	2.28 (0.86)	2.64	0.49
	2	1.33 (0.58)			2.50 (0.79)		
	3	1.45 (0.72)			2.19 (0.92)		
	4	1.36 (0.65)			2.46 (0.78)		
**Nutrition**							
Perceived importance of nutrition to health	1	1.03 (0.43)	1.14	0.33	1.04 (0.47)	1.68	0.17
	2	1.14 (0.59)			0.91 (0.58)		
	3	1.03 (0.47)			1.09 (0.52)		
	4	1.09 (0.56)			1.04 (0.51)		
Awareness about food labels	1	2.14 (0.91)	1.74	0.16	2.80 (0.53)	3.66	0.01
	2	1.95 (0.86)			2.86 (0.46)		
	3	2.20 (0.85)			2.75 (0.60)		
	4	2.27 (0.85)			2.99 (0.12)		
Skill (Interpreting a food label)	1	2.04 (0.93)	2.73	0.04	2.74 (0.61)	2.54	0.06
	2	1.77 (0.82)			2.79 (0.51)		
	3	2.03 (0.90)			2.73 (0.62)		
	4	2.19 (0.87)			2.93 (0.25)		
**Physical Activity**							
Attitude to importance of physical activity for health	1	2.80 (0.57)	0.85	0.47	2.77 (0.60)	0.11	0.95
	2	2.67 (0.74)			2.81 (0.55)		
	3	2.79 (0.59)			2.77 (0.62)		
	4	2.78 (0.60)			2.78 (0.60)		
Awareness about pulse rate	1	1.59 (0.85)	5.26	< 0.01^*^	2.50 (0.82)	6.94	< 0.01^*^
	2	1.34 (0.91)			2.77 (0.57)		
	3	1.58 (0.79)			2.43 (0.85)		
	4	1.88 (0.91)			2.85 (0.49)		
Skill (Measuring pulse rate)	1	1.41 (0.70)	1.33	0.26	2.32 (0.86)	10.77	< 0.01^*^
	2	1.33 (0.61)			2.67 (0.63)		
	3	1.43 (0.66)			2.24 (0.85)		
	4	1.55 (0.80)			2.78 (0.56)		
**Communication**							
Behaviour in communicating about adolescent issues	1	17.06 (5.07)	2.76	0.04	16.78 (4.89)	2.61	0.05
	2	15.97 (4.51)			16.30 (4.42)		
	3	16.67 (4.74)			17.45 (4.71)		
	4	15.20 (5.09)			15.51 (4.97)		

* Statistically significant

### Pretest-posttest HIV/AIDS results across school quintiles

The exposure to HIV/AIDS education in the quintile 4 pretest was significantly different from the other quintiles, however, no significant differences were seen in the posttest. The mean scores for knowledge about HIV/AIDS showed improvement across all quintiles in the posttest. The knowledge about HIV/AIDS, significantly increased for quintile 4, from the pretest (mean 9.76, SD 2.68) to the posttest (mean 11.82, SD 1.32; *p* < 0.01).

### Pretest-posttest obesity results across school quintiles

The mean scores for awareness of obesity and awareness about BMI showed improvement across all quintiles in the posttest. The awareness of obesity, significantly increased for quintile 4, from the pretest (mean 2.22, SD 0.91) to the posttest (mean 2.93, SD 0.30; *p* < 0.01), and quintile 2, from the pretest (mean 1.80, SD 0.92) to the posttest (mean 2.94, SD 0.29; *p* < 0.01).

### Pretest-posttest physical activity results across school quintiles

The mean scores for awareness about pulse rate showed improvement across all quintiles in the posttest. The awareness about pulse rate, significantly increased for quintile 4, from the pretest (mean 1.88, SD 0.91) to the posttest (mean 2.85, SD 0.49; *p* < 0.01).

### Pretest-posttest communication results across school quintiles

In the behaviour in communicating about adolescent issues subscale, although there were no significant differences in the posttest, pretest results showed that quintile 1 students communicated more with their parents than quintile 4.

## Discussion

*The CIrCLE of Life Initiative,* aimed to increase knowledge, improve perceptions and attitudes, shift behaviours related to HIV and obesity among Grade 6 students, and transfer HIV- and obesity-related information from students to parents through shared activities. Positive changes in outcomes were observed across various subscales, such as knowledge about HIV, awareness (obesity, BMI, food labels and pulse rate), and skills (measuring and interpreting pulse rate, interpreting a food label, measuring and interpreting BMI). The statistically significant differences indicate changes 3 months after the intervention in knowledge and behaviours among both students and parents. Other subscales, such as HIV-related attitudes, perception of stigma in the community and attitudes to importance of physical activity for health, showed no statistically significant improvements among either group. Differences were also observed in a few subscales among either students or their parents (but not both). Positive changes among students only included perceptions of stigma in community and exposure to HIV/AIDS education, while positive outcomes observed only among parents subscales included behaviour in communicating about adolescent issues.

The intervention was effective among students in all school quintiles even though there were differences by quintiles in baseline knowledge. In particular, at the pretest, the quintile 4 urban school, which had a higher social-economic status than the other schools, had higher mean scores in some aspects, such as HIV knowledge. Socio-economic variations in the areas in which the schools are located is important to note when selecting priority schools/areas for implementing HIV and obesity prevention interventions. Form an equity perspective targeting lower quintile schools may be desirable. Awareness of how the socio-economic status may affect future implementation efforts is critical within the contextual diversity of different schools. Context differed between fee/non-fee paying schools, urban/rural schools, and diseases, lifestyles, and quality of life experienced by students in the different schools. Understanding the context of intervention delivery is critical to explain outcomes, as different interventions may have other causal processes and relationships within different contexts.

### Knowledge and awareness

The statistically significant pretest-posttest scores suggest that the intervention effectively increased HIV-related knowledge and obesity-related awareness in both students and their parents. According to the educational syllabus, students should be exposed to the HIV/AIDS content, annually, from Grade 4, yet only 37.7% reported being exposed. Many students and parents had reported testing for HIV in the pretests and, in all HIV testing procedures, counselling is a requisite. The results indicate that the education is not being done as required or that students do not experience it as education. The statistically significant increase in HIV-related knowledge in both students’ and parents’ posttests, suggest that *The Circle of Life Initiative* might have been a catalyst in improving knowledge. Obesity awareness, a topic not currently covered in the syllabus, also increased in both groups, likely due to the intervention.

### Attitudes and perceptions

Although the intervention showed positive outcomes in knowledge and awareness in both groups, change in attitudes and perceptions proved more challenging. Like other studies, the programme may have reinforced or validated their concerns, but it did not change their thinking [41]. There were only small shifts in the number of participants that moved between the attitude subscales in the openness about HIV status and the importance of physical activity for health. The intervention positively affected perceptions of stigma in the community among students, but not among their parents. It is unlikely that this school-based intervention alone could influence HIV-related stigma in the community and parents indicated that they would still be apprehensive about openly declaring their HIV status. There is a need for early interventions (Grade six or earlier) to reduce stigma and discrimination among children, especially in schools in rural and impoverished areas [42]. Stigma and discrimination are complex social processes influenced by local contexts and vary widely by school location and socio-economic status [42, 43]. Results from one setting may not translate to others, highlighting the importance of context-specific research and programming.

### Intention to change behaviour

As it is not always possible to measure behaviour change over a short time, the intention to change some behaviours was measured. A longer-term follow-up study would be beneficial to understand the practical impact of the intervention. Parents showed positive shifts in behavioural intention related to HIV testing. Exposure to the intervention could have influenced behavioural intentions. Reported sedentary behaviour also decreased in both groups and sustainability of increased physical activity could be assessed with longer follow up.

There were no statistically significant changes in the perception of the importance of nutrition to health in both students and parents. Parents may be familiar with what constitutes a healthy diet, but many issues influence decision-making and merely providing information may be insufficient [44]. Behaviour occurs in social environments, and the contexts, including political and economic forces, influence people’s health regardless of their nutritional knowledge [44, 45]. The study participants comprised 42.3% unemployed parents higher than the South African national unemployment rate of 27.6% [46]; 77% were dependent on social grants. Even if they wanted to change behaviour, their circumstances were a likely impediment.

### Inter-generational outcomes

Changes were observed in both the student and the parent groups, suggesting that the intervention resulted in inter-generational knowledge transfer. Contrary to the conventional view that parents impart knowledge, values and beliefs to their children [21], our findings indicate that the reverse can also occur, and that parents can learn from their children. Learning might be reinforced through knowledge-sharing and communication within the family structure. About 70% of parents conversed with students about their school day, and about 50% of students sought help with their homework on 4–7 days of the week, presenting an ideal opportunity for inter-generational information transfer. In the posttest, students and parents confirmed that their intention to convey and receive information was high.

The strong link between the classroom and home environments provided a mechanism to transfer information. School-based interventions, planned to extend the programme’s influence beyond the school setting could secure parental participation. Students’ knowledge was communicated to parents verbally, and through activities in the workbook. The process was also supported by the enhancement of existing and new skills.

The effective components of this study can potentially influence school curriculum reform, not only for students but also for parents. The content should be appropriately focused so that activities engage parents. Traditional homework does not emphasise parental engagement. The conventional approach is to ask parents to sign off on completed homework, rather than engage with the content and activities.

### Enhancing skills

Parents have expressed that a lack of knowledge, skills, comfort and confidence limit discussions with their children regarding adolescent issues, sexuality and HIV [27, 47, 48]. Our promising findings suggest that parents will talk to their children if encouraged. Our intervention did not involve direct contact with parents, yet providing activities involving parents facilitated discussions.

Improving parents’ self-efficacy in communication skills proved to be valuable in supporting the quality and quantity of parent pre-adolescent communication [27, 49]. There was an increase in parents’ comfort in communication about adolescent matters, but not among students. Possible reasons that students did not report increased communication about personal issues could include embarrassment when discussing sexual topics; non-conducive environments for open discussions about sexual and reproductive health; and cultural and religious beliefs [27, 50]. The intervention activities created a platform for student-parent interactions, resulting in possible dialogue related to the content.

The programme targeted pre-adolescents in the elementary school phase, and initiated family communication at a stage when parents are still a primary point of attachment. Young people typically begin to engage in risky behaviour in early adolescence [51, 52], so prevention measures may be more effective when targeted in the pre-adolescent phase [41]. For example, early communication - particularly before sexual debut - reduces risky sexual behaviour, potentially reducing the spread of HIV/AIDS [16, 22].

Similar to other studies, learning was heightened when practical and theoretical activities were integrated [21, 27]. The positive changes in some skills suggest that practical activities that involved kinetic learning were important in enhancing theoretical learning. Skills to measure and interpret pulse rate were not previously taught to students, and both groups reported improved skill sets. Parent’s knowledge may also increase as a result of children participating in activities, as they are more likely to discuss new or exciting skills with parents, than written tasks [21].

### Health education in school settings

Schools are critical settings for health education initiatives, and can reach a substantial number of students to foster change [10, 53, 54]. Classroom education often comprises audio and visual communication. The hands-on nature of the intervention was designed to directly and individually involve students through various approaches. The first approach encouraged student creativity, e.g. obesity poster-making and the HIV story-writing activities. The second approach enhanced skills such as, activities measuring and interpreting BMI and pulse rate. Third, the intervention elicited enjoyment and incorporated entertainment through interactive activities and delivery techniques [10]. The multi-component intervention derived its outcomes from the interaction of several components rather than each component fulfilling a separate function. Theme repetition delivered through various modalities, and interrelated messaging, improved receptivity and learning [55, 56].

Implementation of the intervention was not a ‘plug and play’. The needs assessment informed the implementation. For example, educators were appropriately up-skilled to teach health education, transfer health skills, and deal with the psychological aspects of HIV and obesity.

### Strengths and limitations

Strengths included recruiting students from schools representing diverse rural and urban locations, and school quintiles. The high follow-up rate (98% in students and 95% in parents) was noteworthy, considering that parents are often a challenging group to reach [8]. Positive support was also received from educators.

The results of the study should be interpreted in light of several limitations. First, there was no control group with which to compare the pre- and post-test findings. It is possible that the observed improvements in the outcome measures were not a direct result of the intervention and were influenced by other factors, such as the HIV-related activities in the community. Second, the questionnaires elicited self-reported information, which might have introduced information bias, including social desirability and recall biases. Attempts were made to limit the recall biases with careful questionnaire design, and internal validity checks between all student and parent answers. Third, only one parent was recruited per student, which prevented us from examining the complexities of specific family structures and their associated dynamics. Fourth, only the short-term posttest responses (3 months after implementation) were analysed; the longer-term effects of the intervention are unknown.

## Conclusion

*The CIrCLE of Life Initiative,* delivered to pre-adolescent students, with parent participation, could have influenced the changes observed in pretest to posttest scores, specifically knowledge and awareness, self-reported skills and behaviour. However, the intervention had little to no influence on some attitudes and perceptions. The study contributes to the limited literature regarding the transfer of knowledge from children to their parents. With activities that encourage parental engagement, students could influence the knowledge and practices of their parents, supporting inter-generational knowledge and skills transfer. The changes observed could influence the health of both pre-adolescents and their parents, and contribute to sustained effects over time.

## Supplementary Information

**Additional file 1.** TREND Statement Checklist.

## Data Availability

The datasets and materials used and/or analysed during the current study are available from the corresponding author on reasonable request.

## Abbreviations

AIDS: Acquired Immunodeficiency Syndrome
BMI: Body Mass Index
CIrCLE: Child Influencing paRent Communication for Life Education
HIV: Human Immunodeficiency Virus
NCD: Non-Communicable Disease
TREND: A Transparent Reporting of Evaluations with Nonrandomized Designs Statement Checklist
